# Prevalence of psychological symptoms among adults with sickle cell disease in Korle-Bu Teaching Hospital, Ghana

**DOI:** 10.1186/s40359-016-0162-z

**Published:** 2016-11-10

**Authors:** Michael Tetteh Anim, Joseph Osafo, Felix Yirdong

**Affiliations:** 1 Department of Psychological Medicine and Mental Health, School of Medical Sciences, University of Cape Coast, Cape Coast, Ghana; 2 Department of Psychology, University of Ghana, Legon, Ghana; 3 Department of Psychological Medicine and Mental Health, School of Medical Sciences, College of Health and Allied Sciences, University of Cape Coast, Cape Coast, Ghana

**Keywords:** Prevalence, Psychological symptoms, Psychological distress, Sickle cell disease, Chronic disease, Ghana

## Abstract

**Background:**

Previous research revealed high prevalence of psychological symptoms among sickle cell disease (SCD) patients in the West and Europe. In some Black SCD populations such as Nigeria and Jamaica, anxiety and depression had low prevalence rates compared to Europe. With difficulty locating research data on the prevalence of psychological symptoms in Ghana, this study aimed at exploring psychological symptoms among adults with SCD in a Teaching Hospital in Accra, Ghana.

**Methods:**

Two hundred and one participants (males 102 and females 99) who were HbSS (*n* = 131) and HbSC (*n* = 70), aged 18 years and above were purposively recruited. Using the Brief Symptom Inventory (BSI) in a cross-sectional survey, the research answered questions about the prevalence of psychological symptoms. It also examined gender and genotype differences in psychological symptoms scores.

**Results:**

Results indicated that adults with SCD had non-distress psychological symptoms scores. Although paranoid ideation as a psychological symptom indicated “a little bit” score, its prevalence was only 1 %. The prevalence of psychological symptoms as indexed by the Positive Symptom Total (PST) was 10 %. Anxiety, hostility, and depression were psychological symptoms with low scores. Furthermore, except psychoticism scores, males did not differ significantly from females in other psychological symptoms. On the contrary, HbSS participants differed significantly, reporting more psychological symptoms than their HbSC counterparts.

**Conclusions:**

The study concluded that there was low prevalence of psychological symptoms among adults with SCD in this Ghanaian study. Although psychological symptoms distress scores were not observed among study participants at this time, females differed significantly by experiencing more psychoticism symptoms than males. HbSS participants also differed significantly by experiencing more depression, phobic anxiety, paranoid ideation, psychoticism, and additional symptoms such as poor appetite, trouble falling asleep, thoughts of dying, and feeling guilty, than their HbSC counterparts. Implications for further study and clinical practice were discussed.

## Background

SCD is a major genetic disease that negatively impacts individuals in Sub-Saharan African countries [1]. According to the WHO [1] the disease upsets hemoglobin. This results in frequent pain and medical problems that in turn negatively affect the patients’ education, employment, and psychosocial development [1].

The WHO further noted the highest prevalence of hemoglobin AS in Africa as occurring “between latitudes 15^0^ North and 20^0^ South, ranging between 10 and 40 % of the population in some areas. Prevalence levels decrease to between 1 and 2 % in North Africa and less than 1 % in southern Africa” [1]. Ghana, Nigeria, Cameroon, Republic of Congo, and Gabon have prevalence between 20 and 30 % while it is as high as 45 % in some parts of Uganda [1–3].

The reason the sickle cell has maintained such high prevalence levels in tropical Africa is because the sickle cell trait partially protects against malaria [1, 4]. However, individuals who are homozygous for gene S do not have defense against malaria and consequently suffer from severe sickle cell disease, with a lot of them dying before attaining the age of procreation [1]. Such HbSS individuals usually die from an infection or severe anemia [5]. Those who survive into adulthood remain susceptible to exacerbations of the disease and its medical and psychosocial complications [6].

With the present lack of cure, many adults with SCD are believed to live in fear of early death or have death anxiety and many other psychological complications [7]. There are effective treatments using painkillers for the sickle cell pain. Other complications of sickle cell disease are treated using antibiotics. Rest, balanced diet, folic acid supplementation and high fluid intake, plus occasionally needed aggressive procedures like transfused blood and operation are used [8]. However, psychological difficulties accompany these medical complications and treatments. According to Anie [7], Becker, Axelrod, Oyesamni, Markov and Kunkei [9] and Levenson et al. [10], psychological complications and “psychiatric issues are common in sickle cell disease” [11].

Psychological symptoms have been reported in western literature to be highly prevalent among adults with sickle cell disease [10–13]. Depression rates, for example, are comparable to those found in other serious chronic medical diseases. These range “from 18 to 44 %” [11, 14–16]. Depression rates among people living with sickle cell disease are higher than rates in the general population despite controlling for illness-related physical symptoms [11, 17]. Twenty-seven and half percent of adults with sickle cell disease were reported in a PiSCES study as having depression and 6.5 % as having anxiety [10]. The PiSCES project study found that depressed and anxious sickle cell disease persons functioned poorly and used opioids and hospital emergency services frequently [11].

In Africa, however, published literature about psychological symptoms in SCD is scarce. It is only in Nigeria in West Africa that prevalence of specific psychological symptoms in SCD have been reported. Prevalence of depression, for example, was reported higher among sickle cell participants in a Nigerian study than among cancer or malaria study participants. Depression, however, was reported to be lower in persons with SCD than it was in persons living with HIV-AIDs [11, 18]. A similar research studied psychosocial impact of sickle cell disorder in a Nigerian setting. From a sample of 408 adolescents and adults attending three hospitals in Lagos, Nigeria, the authors found depression to be commonly experienced among half of the study participants, while feelings of anxiety and self-hate were uncommon [19].

Since psychological symptoms have implications for physical complaints in sickle cell disease, their study emphasizes the importance of studying psychological symptoms among persons with SCD. The implications are that psychological symptoms are known to contribute to vaso-occlusive crisis and other physical complaints. For example, major depression was reported to increase sickle cell chronic disease patients’ burden of physical illness and symptoms, their functional disabilities and medical costs [20]. Some researchers reported that it is better to consider psychological variables as contributing to the onset of sickle cell pain. For example, Pell and colleagues [21] found that higher levels of kinesophobia were associated with greater psychological distress. Their findings suggest that, it could be psychological distress that increased kinesophobia or kinesophobia increased psychological distress since the analysis was correlational. The psychological symptoms that were associated with higher levels of kinesophobia were Phobic Anxiety, Psychoticism, Somatization, Anxiety, Obsessive-Compulsive, Interpersonal Sensitivity, and depression. Some research found that psychological problems that sickle cell disease patients most frequently encountered are increased anxiety, depression, social withdrawal, aggression, poor relationships, and poor school performance [22].

Elsewhere, it was found that stigmatization in SCD for pseudo-addiction to opioid analgesics was also related to anxiety and depression [10, 23]. Depression was found to powerfully predict physical and mental health-related quality of life than was genotype [10]. Depression in SCD individuals is associated with increased emergency room treatments, hospital admissions, chronic pain flares, SCD crisis, and higher levels of related psychological disorders.

Another importance of examining psychological symptoms is that symptoms of fatigue, appetite disturbance, and irritability are present both in sickle cell anemia and in clinical depression. Patients with the most clinically severe pain also show the greatest prevalence of depression [14, 15].

An association between anxiety, poorer health-related quality of life, and more pain in SCD was established [10]. Therefore, Levenson and colleagues [10] concluded that anxiety and depression predicted more daily pain and poorer physical and mental quality of life in adults with SCD. These findings point out the importance of recognizing and treating psychological symptoms, particularly anxiety and depression, in adults with SCD.

Although it is a challenge determining the exact prevalence rate of a psychological disorder in any given population, some countries as mentioned above have attempted it and have some figures that guide action, policy and research. This is not the case in Ghana which has no records of national statistics on the prevalence rates of psychological symptoms among sickle cell disease patients. Against this background, this study aimed at investigating the prevalence and exploring psychological symptoms among SCD participants in Accra, Ghana.

Subsequently, the following research questions were posed:

1. Is there high prevalence of psychological symptoms among adults with SCD?
2. Is there a significant difference in the mean psychological symptoms score for males and females?
3. Is there a significant difference in the mean psychological symptoms score for HbSS and HbSC persons?

The study was conceptualized based on the existing theoretical view that women experience and display more psychological symptoms than males [24] and HBSS persons experience more pain and severe psychological distress than HBSC persons [5, 25–27].

This prevalence study was necessary because the lack of baseline data on psychological symptoms makes providing specific psychological services to sickle cell disease individuals uncertain. The study contributed knowledge of prevalence of psychological symptoms from the Ghanaian experience. It contributed information that is lacking on gender and genotype differences in the experience of psychological symptoms. The study firmed up the fact about the low prevalence of psychological symptoms among adults with SCD who live in non-westernized countries as against the high prevalence among those who live in highly industrialized countries. It hints about possible geographical and socio-cultural factors that differentiate psychological symptoms prevalence rates across the world.

## Method

### Participants

Two hundred and one adults with SCD were recruited from the sickle cell clinic at the Korle-Bu Teaching Hospital, Accra, Ghana. The GPower software program was used to determine an adequate sample size to reduce risk of type 2 error. One hundred and seventy-five participants were adequate to recruit for the study. But the researchers contacted 229 potential participants to allow for missing questionnaires and make up for those who would withdraw or not consent. Nine potential participants did not consent, and 19 either poorly completed the questionnaires or did not return them. Study participants were selected from a total of about 11, 230 patients (Korle-Bu Sickle Cell Clinic records, 2014). The 201 study participants comprised 103 males (51 %) and 99 females (49 %). Participants with HBSS genotype represented 65 % and HBSC represented 39 %. They were sampled purposively to satisfy inclusion criteria of having either HBSS or HBSC, of being 18 years and above and being able to read and understand English. They were not in crisis and gave both verbal and written consent to participate in the study.

### Research design

The cross-sectional survey design was used for the study. This was the most appropriate design according to Smith and Davis [28] to survey the opinions of persons of interest at a point in time. It is also the most appropriate design for conducting prevalence studies [29].

### Measuring instruments and materials

The participants completed demographical data about their gender, age, marital status, and education, and their medical records were consulted to confirm their sickling diagnosis. The Brief Symptom Inventory (BSI) [30] is a 53-item instrument that measures psychological symptoms. Respondents rated items on a five-point scale from 0 representing “not at all” to 4 representing “extremely.” The BSI has nine subscales that include Somatization, Obsession-Compulsion, Interpersonal Sensitivity, Depression, Anxiety, Hostility, Phobic Anxiety, Paranoid Ideation, and Psychoticism.

The BSI showed suitable reliability and validity values. It has internal consistency alpha coefficients that range from 0.71 to 0.85 for the nine symptoms subscales. It has test-retest reliability coefficients that range from 0.68 (Somatization) to 0.91 (Phobic Anxiety) [30].

To measure overall psychological distress, the Global Severity Index (GSI) was used [30]. The GSI is a measure of the average of psychological distress scores on all nine symptoms. Higher scores show that psychological distress is greater. Test-retest reliability for the three Global Indices ranged from .87 (PSDI) to .90 (GSI). In this current study, Cronbach’s alpha coefficients for all nine subscales were Somatization (.70), Obsession-Compulsion (.70), Interpersonal Sensitivity (.68), Depression (.74), Anxiety (.69), Hostility (.69), Phobic Anxiety (.63), Paranoid Ideation (.73), and Psychoticism (.69). The whole scale had a Cronbach’s alpha coefficient of .94 which was good, making the scale reliable for use to collect data from this Ghanaian SCD sample.

### Procedure

After ethical clearance was obtained from the Noguchi Memorial Institute for Medical Research Institutional Review Board, permission was obtained from the Director of the Institute of Clinical Genetics, Korle-Bu Teaching Hospital to collect survey data from the sickle cell clinic in Korle-Bu Teaching Hospital. Written informed consent was obtained from potential participants and the principal researcher and his assistants handed the questionnaires to SCD participants who awaited their turn to see their doctor. The questionnaires were completed and collected same day before participants left the clinic. About nine potential participants refused to give consent and 19 did not return the questionnaires, or filled them poorly or returned uncompleted questionnaires. Return rate was 91 %, that is, 201 questionnaires were retrieved out of 220.

#### Data analysis

Descriptive statistics, calculation of prevalence rate, and independent samples t-tests were used to answer the research questions.

Prevalence rate is a ratio of the number of present cases of a disorder to the number of potential cases [31]. It is the total number of cases existing in a defined population at a specific time, generally measured by doing a survey [28, 31]. To calculate the prevalence rate, the number of individuals in the population who have an illness (e.g. depression) is divided by the total population at risk for the illness.

$$ \mathrm{Prevalence}=\mathrm{Number}\kern0.3em \mathrm{of}\kern0.3em \mathrm{cases}\kern0.3em \mathrm{at}\kern0.3em \mathrm{one}\kern0.3em \mathrm{time}\kern0.3em \mathrm{point}\div $$

Total number of individuals in the defined population at same time point.

As shown by the formula, prevalence is a proportion and can never be greater than one [32]. Specific prevalence measures include point prevalence and period prevalence where point prevalence is the number of individuals who have an illness at a specific point in time divided by the total population who could potentially have an illness on that date. Period prevalence is the number of individuals who have an illness during a specific time period divided by the total population who could have the illness midyear in that year [32].

## Results

To answer the first research question, descriptive statistics such as means and standard deviations were calculated for the scale and subscale scores of the Brief Symptom Inventory (BSI). The results revealed a non-distress score for the Global Severity Index [GSI] (*m* = .18, *SD* = .14). This score is a measure of general psychological distress level. Additionally, all the subscale scores, namely, somatization, obsessive-compulsive, depression, anxiety, hostility, phobic anxiety, and psychoticism indicated non-distress scores except paranoid ideation (m = 1.01, SD = .86) which indicated “a little bit” psychological distress score. A score that is less than 1 is considered by the scale authors as non-distress score (Table 1).

**Table 1:** Summary of Descriptive Statistics Showing Prevalence of Psychological Symptoms

Variable (*n* = 201)	Minimum	Maximum	Mean	Std Dev	Prevalence
Somatization	.01	3.29	.86	.68	0.00
Obsessive-compulsive	.01	3.01	.80	.69	0.00
Interpersonal sensitivity	.01	3.76	.97	.85	0.00
Depression	.01	2.84	.65	.72	0.00
Anxiety	.01	3.01	.59	.62	0.00
Hostility	.01	4.00	.64	.71	0.00
Phobic Anxiety	.01	3.01	.49	.63	0.00
Paranoid Ideation	.01	3.80	1.07	.86	0.01
Psychoticism	.01	3.01	.52	.62	0.00
PST	.00	45.00	19.77	11.03	0.10
PSDI	.01	5.01	1.81	.71	0.01
Additional Items	.00	11.00	2.86	2.80	-
GSI	.01	.63	.18	.14	0.00

Source: Field data, 2014

Scale score: 0 = not at all; 1 = a little bit; 2 = moderately; 3 = quite a bit; 4 = extremely

To calculate the prevalence rate of psychological symptoms among SCD participants, the mean number of individuals in the SCD sample who had indicated non-zero responses and thus revealed the number of symptoms the respondent reported experiencing, was divided by the total population at risk for the psychological symptom (*n* = 201). Thus, 19.77 divided by 201 SCD participants equaled 0.10 (10 %) [Table 1].

To answer the second research question, an independent samples t-test was conducted to compare the psychological symptoms (BSI and subscales) scores for males and females. The results indicated significant mean difference occurring only in psychoticism subscale scores for males (*m* = .43, *SD* = .55) and females (*m* = .62, *SD* = .66); *t* (199) = −2.12, *p* = .03, two-tailed. The magnitude of the differences in the means (mean difference = −.18, 95 % *CI*: −.35 to −.01) was very small [eta squared = 0.02] (Table 2).

**Table 2:** Summary Table of independent t- Test result showing mean comparison of males and females on BSI and subscale scores

Variables	Mean (SD)		Mean diff	F	T	Df	Sig (2-tailed)	Eta sq.
	M (*N* = 102)	F (*N* = 99)						
Somatization	.77 (.65)	.95 (.69)	−.18	.654	−.1.89	199	.06	
Obsessive-compulsion	.77 (.69)	.83 (.71)	−.06	.780	−.66	199	.51	
Interpersonal sensitivity	.90 (.89)	1.03 (.81)	−.12	.234	−1.02	199	.30	
Depression	.57 (.65)	.73 (.77)	−.15	6.317	−1.54	199	.12	
Anxiety	.52 (.58)	.67 (.64)	−.15	.894	−1.70	199	.09	
Hostility	.68 (.82)	.60 (.59)	.07	2.837	.72	199	.47	
Phobic Anxiety	.42 (.61)	.57 (.64)	−.15	1.048	−1.70	199	.09	
Paranoid Ideation	1.02 (.87)	1.12 (.84)	−.10	.222	−.82	199	.41	
Psychoticism	.43 (.55)	.62 (.66)	−.18	9.266	−2.12	199	.03	.02
Additional Items	2.57 (2.65)	3.15(2.93)	−.57	.659	−1.46	199	.14	
BSI	.17 (.13)	.19 (.13)	−.03	.501	−1.58	199	.11	

*P* < 0.05

To answer research question three, an independent samples t-test was conducted to compare the psychological symptoms (BSI and subscales) scores for HbSS and HbSC genotypes. The results indicated that there was significant difference in GSI scores for HbSS (*m* = .20, *SD* = .14) and HbSC (*m* = .15, *SD* = .13); *t* (199) = 2.42, *p* = .01, two-tailed. The magnitude of the differences in the means (mean difference = .05, 95 % *CI:* .01 to .09) was small [eta squared = 0.03] (Table 3). Similarly, significant differences were indicated in four subscales, namely, Depression, Phobic Anxiety, Paranoid Ideation, and Psychoticism, together with significant differences in Additional Items (i.e., Poor Appetite, Trouble Falling Asleep, Thoughts of Death or Dying, and Feeling of Guilt).

**Table 3:** Summary Table of independent t- Test result showing mean comparison of HbSS and HbSC on BSI and subscale scores

Variables	Mean (SD)		Mean diff	F	T	Df	Sig (2-tailed)	Eta sq.
	HbSS (*N* = 131)	HbSC (*N* = 70)						
Somatization	.88 (.67)	.81 (.68)	.07	.100	.76	199	.44	
Obsessive-compulsion	.85 (.71)	.69 (.67)	.16	.542	1.59	199	.11	
Interpersonal sensitivity	1.05 (.85)	.81 (.83)	.24	.389	1.89	199	.05	0.02
Depression	.72 (.75)	.51 (.63)	.21	4.185	1.98	199	.04	0.02
Anxiety	.59 (.57)	.58 (.68)	.01	.786	.18	199	.85	
Hostility	.71 (.75)	.51 (.62)	.20	3.908	1.87	199	.06	
Phobic Anxiety	.56 (.67)	.36 (.50)	.20	4.576	2.17	199	.03	0.02
Paranoid Ideation	1.20 (.88)	.80 (.74)	.40	3.252	3.26	199	.00	0.05
Psychoticism	.59 (.62)	.40 (.59)	.19	1.716	2.12	199	.03	0.02
Additional Items	3.16 (2.89)	2.30 (2.52)	.86	3.943	2.09	199	.03	0.02
BSI	.20 (.14)	.15 (.13)	.05	1.167	2.42	199	.01	0.03

*P* < 0.05

## Discussion

It was the objective of this study to determine the prevalence rate of psychological symptoms among adult participants with SCD, and to examine gender and genotype differences in psychological symptoms in this sample. First, the main findings indicated that 10 % of Positive Symptom Total (PST) was prevalent among the sampled SCD participants. Since PST is a count of all the items with non-zero responses and reveals the number of symptoms the respondents report experiencing [30], we conclude that 10 % of psychological symptoms were present at this time. This notwithstanding, participants’ overall psychological distress was 0.00; neither did they have specific psychological symptoms except paranoid ideation that recorded a one percent prevalence rate. Phobic anxiety, anxiety, hostility and psychoticism were among subscales that recorded low mean rates, suggesting that they were least experienced.

This result is comparable to others recorded in Nigeria and Jamaica. The Nigerian study indicated that anxiety was almost absent among a group of adults with SCD but not depression [18]. Thomas, Hambleton, and Sergeant [33] found that Jamaican patients (*n* = 50) with homozygous SCD had less general anxiety, a lower emotional response to pain, and lower levels of perceived pain compared to their London counterparts (*n* = 50) who believed the disease had a more marked effect on their psychological health.

The results are however dissimilar to psychosocial findings among United Kingdom and United States of America SCD participants where high incidence and prevalence of psychological symptoms are reported among participants with SCD [10, 12, 13, 33, 34]. Levenson and colleagues [10] found a high prevalence of about 28 % of depression among African Americans. Hasan, Hashmi, Alhassen, Lawson and Castro [14] found depression ranging from 18 to 44 % among SCD participants comparing their rates to the general population.

For some African countries and continental Black SCD populations to indicate low rates of depression, anxiety, and hostility, suggests that culture might play some role in the coping and psychological functioning of adult SCD individuals. This study did not investigate the role of culture in the psychological functioning of SCD participants. But it is highly possible there are cultural differences in the coping and psychological functioning of SCD individuals. Bediako [35] and Barbarin and Christian [36] alluded to this.

Second, males and females did not differ significantly in their experience of psychological symptoms. There were no previous studies to compare this result with since previous sickle cell research did not compare males and females on psychological symptoms. In this study, however, males and females alike had low psychological symptoms scores either in their global severity index score or on each of the symptoms subscales, except on the psychoticism subscale on which females (*m* = .62, *SD* = .66) and males (*m* = .43, *SD* = .55) recorded significant difference, *t* (199) = −2.12, *p* = .03, eta squared = .02. By this result, female adults with SCD reported more psychoticism symptoms than male adults with SCD. Psychoticism items were indicative of a withdrawn, isolated, schizoid lifestyle. The subscale provides for a graduated continuum from mild interpersonal alienation to dramatic psychosis, as defined by Eysenck and Eysenck. These are the symptoms the study result indicated that the female SCD participants experienced more than the males.

According to Colman [37], psychoticism is a psychological condition or state characterized by psychosis or traits such as aggressiveness, coldness, impulsiveness, antisocial behavior, tough-mindedness, and creativity. We opine that these Ghanaian adult SCD females used psychoticism as a defense mechanism to cope with SCD, and not that they experienced psychosis which is a mental disorder characterized by delusions and/or prominent hallucinations without insight into their pathological nature. This is because these SCD females did not exhibit mental impairments that grossly interfered with their capacity to meet ordinary demands of life. Forty-eight percent of them were students and 43 % of them were employed in public, private and personal occupations, suggesting that they led meaningful lives and contributed to their communities. They were thus psychologically healthy according to the European Commission’s [38] description of psychological health.

HbSS participants significantly differed from HbSC participants on psychological symptoms. Similar to previous research, HbSS participants outnumbered HbSC participants in any research sample and also experienced more symptoms than HbSC participants. Given that HbSS is a more severe form of the disease than the HbSC genotype [5, 25], and given that disease severity determines the degree of psychological symptoms the patient experiences [25, 27], it is not surprising this current finding agrees with previous research. The severer the disease, the more psychological symptoms the patient reports or experiences. This is consistent with the body- mind interaction and relationship, that whatever happens to the body affects the mind, and whatever happens to the mind affects the body in similar proportion [31].

Furthermore, it is observed from Table 3 that there were significant genotype differences in the following subscales, namely, Depression, Phobic Anxiety, Paranoid Ideation, and Psychoticism, with HbSS participants reporting significantly higher mean scores than HbSC participants. This implies that HbSS participants experienced more of these symptoms than their HbSC counterparts, even if they experienced them to a degree that did not reach distress levels as previously noted. Additionally, HbSS participants experienced significantly more symptoms such as poor appetite, trouble falling asleep, thoughts of dying, and feelings of guilt than their HbSC counterparts.

Because no previous published literature have been found to compare the above-mentioned findings, we consider that these are new findings from Ghana that add up to the existing literature and fill some literature gap.

## Limitations and future research

Although the results are useful in giving some idea about prevalence of psychological symptoms, their practical use should be cautioned. This is because, the analyses were not done based on results of principal component analysis (PCA) or confirmatory factor analysis (CFA) of the Brief Symptom Inventory. Although content validity was assured, it was not enough. Future research should consider factor analyzing the scale by using PCA and CFA technique to ascertain the scale’s utility for the Ghanaian sample to further determine if the scale and subscales are both valid and reliable.

Given that the results cannot be generalized beyond adult SCD participants in the sickle cell clinic in Korle-Bu Teaching Hospital in Accra, Ghana, replication of the study using other SCD and other chronic disease populations is highly recommended.

The study could not tell what accounted for the non-distress psychological symptoms scores among participants. It is recommended that future study should consider testing a theory involving psychological symptoms or health to ascertain the possible cause of non-distress psychological symptoms scores among SCD participants. Additionally, there were no other groups in the study with which to compare the prevalence rates, (i.e., whether SCD participants experience non-distress psychological symptoms scores compared with other chronically ill populations and the general healthy population. Further research in this direction is encouraged.

One more limitation was the cross-sectional design that was used where the researchers could not determine how particular individuals developed over time because the researchers did not follow up on individuals. A longitudinal study would have permitted observation of individuals over time.

## Conclusion

The research concludes that there is high probability of low prevalence rate of psychological symptoms. Majority (90 % ) of adult SCD study participants in the sickle cell clinic at the Korle-Bu Teaching Hospital, Accra, Ghana, had non-distress psychological symptoms scores in Anxiety, Depression, Hostility, Phobic Anxiety, Psychoticism, Interpersonal Sensitivity, Somatization, and Obsessive-Compulsion. Although psychological symptoms distress scores were not observed among study participants at this time, females differed significantly by experiencing more psychoticism symptoms than males. HbSS participants also differed significantly by experiencing more depression, phobic anxiety, paranoid ideation, psychoticism, and additional symptoms such as poor appetite, trouble falling asleep, thoughts of dying, and feeling guilty, than their HbSC counterparts.

The main importance of this prevalence estimates is to gain an understanding of the percentage of individuals in the sickle cell disease population at the Korle-Bu Teaching Hospital who remain psychologically functional after having received a diagnosis and live with sickle cell disease. Such statistics should be useful to the sickle cell clinic charged with planning for the provision of health, continuing medical consultations, and psychological services, and for long-term counselling and support.

Second, this prevalence study is of interest because it forms part of the process of systematically assessing the reality of psychological symptoms under surveillance [39]. It provides information that points toward psychological areas that may require further attention. This study provides baseline data on which to base future assessments of changing patterns by means of assessments performed over time. According to Boyle [40] and Silver, Ordunez, Rodriguez, and Robles [41], prevalence studies are useful for quantitatively and qualitatively assessing changes that take place. This feature makes prevalence studies potential instruments for evaluation purposes.

The statistic on prevalence of psychological symptoms found in this study would be most useful in assessing the impact of psychological symptoms on adults with SCD in the Korle-Bu Teaching Hospital’s sickle cell clinic, their families, and the society. The results would be useful in planning for healthcare services. This statistic would help the sickle cell clinic management do some accurate healthcare-related needs assessments. The results further suggest that the management, staff, and clinicians have been offering some services that accrue to patients’ psychological benefit. These psychologically beneficial factors in the clinic must be investigated for the purpose of emphasizing and maintaining them.

The psychologist can be certain and confident about how much psychological intervention is needed in the team management effort of the clinic and where to place emphasis in providing psychological services to SCD patients. The purpose, degree and target of psychological intervention become clearer. In light of the results, the management objective for now might be to emphasize positive psychology among patients and not psychotherapy.

## References

[1] WHO Regional Office for Africa (2015). Sickle cell disease prevention and control.

[2] Centres for Disease Control and Prevention (2012). Sickle cell disease data and statistics.

[3] Loureiro MM, Rozenfield S (2005). Epidemiology of sickle cell disease hospital admissions in Brazil. Rev Saude Pubica.

[4] Modell B, Darlison M (2008). Global epidemiology of haemoglobin disorders and derived service indicators. Bull World Health Organ.

[5] Konotey-Ahulu FID (1991). The sickle cell disease patient.

[6] Quinn CT, Rogers ZR, McCavit TL, Buchanan GR (2010). Improved survival of children and adolescents with sickle cell disease. Blood.

[7] Anie AA (2005). Psychological complications in sickle cell disease. Br J Haematol.

[8] de Montalembert M (2008). Management of sickle cell disease. BJM Clin Rev.

[9] Becker M, Axelrod DJ, Oyesanmi O, Markov DD, Kunkel EJ (2007). Hematologic problems in psychosomatic medicine. Psychiatr Clin North Am.

[10] Levenson JL, McClish DK, Dahman BA (2008). Depression and anxiety in adults with sickle cell disease: the PiSCES project. Psychosom Med.

[11] Levenson JL. Psychiatric Issues in Adults with Sickle Cell Disease. Primary Psychiatry, 2008. http://primarypsychiatry.com/psychiatric-issues-in-adults-with-sickle-cell-disease/. Accessed 7 Jul 2016.

[12] Alao AO, Cooley E (2001). Depression and sickle cell disease. Harv Rev Psychiatry.

[13] Alao AO, Dewan MJ, Jindal S, Effron M (2003). Psychopathology in sickle cell disease. West Afr J Med.

[14] Hasan SP, Hashmi S, Alhassen M, Lawson W, Castro O (2003). Depression in sickle cell disease. J Natl Med Assoc.

[15] Laurence B, George D, Woods D (2006). Association between elevated depressive symptoms and clinical disease severity in African-American adults with sickle cell disease. J Natl Med Assoc.

[16] Wison Schaeffer JJ, Gil KM, Burchinal M (1999). Depression, disease severity, and sickle cell disease. J Behav Med.

[17] Molock SD, Belgrave FZ (1994). Depression and anxiety in patients with sickle cell disease: conceptual and methodological considerations. J Health Soc Policy.

[18] Ehigie BO (2003). Comparative analysis of the psychological consequences of the traumatic experiences of cancer, HIV/AIDS, and sickle cell anemia patients. IFE Psychologia.

[19] Anie KA, Egunjobi FE, Akinyanju OO (2010). Psychosocial impact of sickle cell disorder: perspectives from a Nigerian setting. Glob Health.

[20] WHO. Sickle cell disease and other haemoglobin disorders. Fact sheet N°308 January 2011. www.who.int/entity/mediacentre/factsheets/fs308/en/. Accessed 3 June 2013.

[21] Pells J, Edwards CL, McDougald CS, Wood M, Backsdale C, Jonassaint J, Leach-Beale B, Byrd G, Mattis M, Harrison M, Feliu M, Edwards L, Whitfield K, Rogers L (2007). Fear of movement (Kinesiophobia), Pain, and Psychopathology in Patients with Sickle Cell Disease. Clin J Pain.

[22] Anie KA, Green J. Psychological therapies for sickle cell disease and pain. Cochraine Database Syst Rev. 2012;2:CD001916. The Cochraine Collaboration. Issue 2, John Wiley and Sons, Ltd. Accessed at http://www.thecochrainelibrary.com.

[23] Elander J, Lusher J, Bevan D, Telfer P, Burton B (2004). Understanding the causes of problematic pain management in sickle cell disease: evidence that pseudo-addiction plays a more important role than genuine analgesic dependence. J Pain Symptom Manag.

[24] Hurtig AN (2008). Relationships in families of children and adolescents with sickle cell disease. J Health Soc Policy.

[25] Platt OS, Brambilla DJ, Rosse WF (1994). Mortality in sickle cell disease. Life expectancy and risk factors for early death. N Engl J Med.

[26] Nettles AS (2008). Scholastic performance of children with sickle cell disease. J Health Soc Policy.

[27] Scott KD, Scott AA (1999). Cultural therapeutic awareness of sickle cell anemia. J Black Psychol.

[28] Smith RA, Davis SF (2004). The psychologist as a detective.

[29] Schneider M (2014). Introduction to public health.

[30] Derogatis LR (1993). Brief Symptom Inventory (BSI): Administration, scoring and procedures manual.

[31] Fadem B (2004). Behavioural science in medicine.

[32] Carneiro I, Howard N (2011). Introduction to epidemiology.

[33] Thomas VJ, Hambleton I, Serjeant G (2001). Psychological distress and coping in sickle cell disease: comparison of British and Jamaican attitudes. Ethn Health.

[34] Anie KA, Dasgupta T, Ezenduka P, Anarado A, Emodi I (2007). A cross-cultural study of psychosocial aspects of sickle cell disease in the UK and Nigeria. Psychol Health Med.

[35] Bediako SM, Neville HA, Tynes BM, Utsey SO (2009). Psychosocial aspects of sickle cell disease: A primer for African American psychologists. Handbook of African American psychology.

[36] Barbarin OA, Christian M (1999). The social and cultural context of coping with sickle cell disease: I. A review of biomedical and psychosocial issues. J Black Psychol.

[37] Colman AM (2009). Dictionary of psychology.

[38] European Commission Green Pape (2005). Improving the mental health of the population: Towards a strategy on mental health for the European Union, Health & Consumer Protection Directorate-General, Brussel.

[39] Creary M, Williamson D, Kulkarini R (2007). Report from the SCD: current activities, public health, implications and future directions. J Womens Health.

[40] Boyle MH (1998). Guidelines for evaluating prevalence studies. Evid Based Mental Health.

[41] Silva LC, Ordúñez P, Rodríguez MP, Robles S (2001). A tool for assessing the usefulness of prevalence studies done for surveillance purposes: the example of hypertension. Rev Panam Salud Publica/Pan Am J Public Health.

